# “Some Kind of Magic?” How Adaptive Experts Navigate Complexity in Pediatric Ultrasound-Guided Vascular Access

**DOI:** 10.5334/pme.1798

**Published:** 2025-11-12

**Authors:** Martien H. Humblet, J. Mesman, L. Lingard, J. Frèrejean, W. N. K. A. van Mook, P. L. J. M. Leroy

**Affiliations:** 1 Department of Pediatrics, Division of Pediatric Intensive Care, Maastricht University Medical Center MUMC+, The Netherlands; 2 School of Health Professions Education, Maastricht University, The Netherlands; 3 Faculty of Arts and Culture, Department of Technology and Society Studies, University Maastricht, The Netherlands; 4 Maastricht University Science, Technology and Society Studies, Maastricht University, The Netherlands; 5 Centre for Education Research & Innovation, Department of Medicine, Schulich School of Medicine & Dentistry and Faculty of Education, Western University, London, Canada; 6 Faculty of Health, Medicine and Life Sciences, School of Health Professions Education, Maastricht University, The Netherlands; 7 Department of Intensive Care Medicine, and Academy for Postgraduate Medical Training, Maastricht University Medical Center MUMC+, The Netherlands

## Abstract

**Introduction:**

Technological innovations hold great promise for enhancing clinical practice, especially in high-stakes settings. Although simulation-based education can help to develop skill proficiency, transferring skills into real-world high-stakes settings remains challenging. By investigating ‘super-users’—exceptional performers who have successfully implemented new technologies in demanding contexts—this study zooms in on ultrasound-guided vascular access (UGVA) in young, awake, and often non-compliant children and aims to unravel complexities and strategies for successful performance in complex contexts.

**Methods:**

Using a constructivist grounded theory approach, sensitized by concepts from expertise theory, we conducted incident-based interviews with 11 experts in pediatrics.

**Results:**

Two main results were identified through theoretical sampling, thematic and conceptual analysis, and constant comparison within a multidisciplinary research team. First, participants described multiple, intersecting dimensions of complexity: conceptual, psychomotor, contextual, and educational. Second, experts articulated their largely tacit knowledge and collaborative dynamics for navigating these complexities metaphorically. Successful pediatric UGVA was likened to a “choreography”, where “orchestration” of dynamic socio-material aspects was perceived to yield “some kind of magic” engagement and synergy of the collective.

**Discussion:**

This study’s conceptualization of dynamic, high-stakes, and morally charged performance reflects adaptive expertise and illustrates how motor skill expertise is intertwined with cognitive, social, and contextual aspects. Hence, in helping to demystify the “magic” in expert performance, this work discusses insights to advance adaptive expertise theory and motor learning theory, particularly on how to prepare future adaptive experts for the inherent complexities and transfer challenges of procedural performance in clinical practice. The findings underscore the need for training approaches that move beyond routine skill acquisition towards dynamic, context-rich learning environments that foster perceptual-motor adaptability, collaborative coordination, and moral readiness for high-stakes performance.

## Introduction

Technological innovations in healthcare aim to enhance clinical performance and often offer the greatest promise in high-stakes settings [1,2,3]. However, training to effectively apply these technologies in such complex settings remains a significant challenge [2,4]. For example, point-of-care ultrasound (POCUS) – a complex skill with high psychomotor demands – is widely recognized as a valuable technological tool to improve rapid diagnostic decision-making and procedural performance, particularly in acute care settings [5]. However, despite its potential, POCUS competency development and transfer across diverse clinical practices appear to vary significantly [5,6]. Current training – typically consisting of didactics and hands-on simulation – is often perceived as insufficiently aligned with the realities of clinical practice [5,6]. Similar complex skills that demand fluid performance under pressure – such as video-laryngoscopy or regional anesthesiology – also face challenges in translating training into reliable real-world clinical performance [7,8]. Developing or adapting training programs for such contexts remains difficult without a deeper understanding of the factors that influence performance in high-stakes contexts [9,10,11,12]. As such, POCUS exemplifies a broader issue in health care and complex skill learning: although new skills can often be performed proficiently in simulation-based training, their effective application in complex, high-stakes contexts remains elusive [5,13,14,15,16].

This transfer challenge has prompted decades of interdisciplinary research and the development of various accounts on transfer [17]. The predominant account, rooted in education and cognitive psychology, conceptualizes transfer as the cognitive process of retrieving and applying previously acquired knowledge from one context to solve problems in another [13,17]. Medical educational scholars have raised concerns that current transfer research often treats context as a variable to control or neutralize in the pursuit of generalizable findings [12]. This decontextualized view overlooks how deeply transfer depends on context, especially in high-stakes settings where no two situations are alike [12,18]. In parallel, extensive motor control and learning theory offer rich insights into precise motor skill acquisition and transfer [19,20,21,22]. Yet, much of this work is based on kinematic data in controlled settings, potentially overlooking the influence of context [19,22]. So, where traditional motor learning theories – such as Fitts and Posner’s stages of skill acquisition – focus on mastery of isolated motor movement [21], more modern perspectives like multiple process models or ecological dynamics emphasize the inseparable relationship between the perceptual motor task, environment, and performer [23,24]. Together, these perspectives resonate with instructional design and learning theory that argue that learning and transfer are optimized when known skills and knowledge are embedded in meaningful authentic contexts [25,26].

Therefore, with the premise that transfer cannot meaningfully be understood without accounting for context, alternative qualitative research designs may complement current academic discourse by exploring the complex interplay between skill and context [27]. While case studies may offer in-depth insights to unravel this phenomenon, they may also face challenges to ensure generalizability to the broader medical field [27]. Consequently, scholars increasingly advocate for theoretically grounded and pluralistic approaches to capture the dynamic interactions that occur in real-world clinical complexity [11,27,28,29]. This may advance our understanding of real-world performance and its inherent complexity, which remain relatively under-theorized, yet are increasingly important for informing education in today’s era of rapid technological enhancements and growing health care complexity [11,28].

One possible approach involves investigating expert performers – in technology literature also termed “super-users” – who have bridged the transfer gap and successfully implemented new technological innovations in complex settings [30,31]. This focus on expert performance at the intersections of the technical and medical domains aligns with expert performance theory, which seeks to explain how individuals achieve expert-level performance in complex tasks [2,32]. In today’s increasingly technology-driven medical landscape, there is a call to reevaluate how real-world expertise develops [33] and what technology-enhanced performance entails at the highest levels of complexity [2].

Responding to the calls for understanding complexity of high-stakes, technology-enhanced performance and real-world motor skill acquisition in medicine, this study zooms in on ultrasound-guided vascular access (UGVA) in complex pediatric contexts. UGVA is a procedural POCUS application that promises to improve pediatric difficult intravenous access (DIVA) [34,35]. Yet, it is highlighted as a complex bimanual technique that requires precise eye-hand-needle coordination for real-time dynamic needle tip visualization and placement within the target blood vessel [36]. While UGVA has become the gold standard for central vascular access in deeply sedated children within pediatric intensive care settings, its adoption in contexts of neonates and non-ICU settings is challenging, despite recognized benefits and the recognized high stakes in this DIVA subgroup [5,35,37]. The subgroup of young and awake children and neonates faces the most risk for DIVA due to both anatomical constraints and the limited ability to cooperate, often leading to delays in necessary treatment and significant psychological trauma from repeated failed attempts [35,38]. Although studies point to the potential benefits of UGVA, its superiority remains inconclusive—largely attributed to the high level of expertise it demands [35,39]. Particularly in the subgroup of young and awake children, the demands of real-world performance may exceed the outcomes of current training approaches [31,40]. To the best of our knowledge, no research has yet examined the specific factors contributing to the recognized complexity in this subgroup [40], the proficiency levels required for success [39], or the reasons behind the consistent success of recognized ‘super-users’ [31,41].

Given the potential impact of more efficient training programs for UGVA in pediatrics, this study aims to explore expert performance of the UGVA technology in young and awake children and neonates. By unravelling its complexities, we aim to understand how expert performers navigate these complexities in successful performance. In doing so, we seek to advance a broader educational discourse to help technology-enhanced clinical performance reach its potential in complex contexts and support expertise development in the workplace.

## Methods

### Study Design

This study employed constructivist grounded theory (CGT) to explore the complexity of contextual motor skill performance [42]. CGT is effective for conceptualizing social phenomena in real-world contexts that are not fully understood. Conceptual insights unfold iteratively through data collection, thematic and conceptual analysis, constant comparison, and theoretical sampling [29,43]. CGT integrates sensitizing concepts from relevant theories, thereby connecting the work to existing knowledge and offering the opportunity to refine that theoretical understanding [29]. We drew on three concepts from expertise theories [44]. First, when aiming to improve educational outcomes, Ericsson’s *expert performance approach* argues to identify and strive for reproducible superior performance as a final goal of training [32]. This concept has directed our study design and sampling of expert performers. Secondly, it is recognized that experts may unintentionally omit information that has become automated or tacit, especially in motor skill performance [45,46]. This influenced our choice of interview techniques that *elicit experts’ tacit cognitive processes* [47]. Thirdly, our understanding that expertise is inherently linked to context directed our attention to *the social dimensions of expertise* and how it interacts with the procedural domain in the “messiness” of real-world performances [48].

The study was approved by both local and national Ethics Review Committees (METC 2021–2656 – NERB 2022.6.1).

### Research team and Reflexivity

CGT recognizes that meaning is co-constructed and, thus, researchers’ orientations shape the work.

A multidisciplinary research team with different epistemological perspectives was chosen to enrich the work, as we negotiated cognitive and constructivist framings of this research. MH and PL are staff members of a pediatric intensive care unit with clinical and educational expertise relevant to UGVA and procedural comfort in children. JF is trained as a cognitive psychologist with expertise in instructional design; JM is a social scientist with qualitative expertise in studying medical practices; LL is an educational researcher expert in constructivist grounded theory methodology and the study of team processes; and WvM is an intensivist and educational leader with a focus on professionalism.

### Sampling strategy

We sought to sample experts in UGVA in children. In the absence of formal certification of such expertise, we identified participants through chain referral sampling with two inclusion criteria: participants had to (1) meet the definition of “subject matter experts” through extensive experience and consistent success and (2) be recognized by peers as the ‘go-to’ person for UGVA in children in their setting [45]. Eligibility was verified by the primary investigator (MH) with clinical expertise in UGVA. Theoretical sampling to the point of sufficient understanding of key themes guided iterative data collection and analysis [29]. (Supplementary file: Table)

### Data collection and analysis

To elicit tacit cognitive processes, interviews were based on incident-based techniques [47]. Every interview started by asking participants to recall memorable clinical cases when UGVA in a child was particularly challenging or successful. We then probed their cognitive processes regarding (A) stepwise approaches, (B) complexity factors, and (C) practice variations [49]. Finally, we explored skill acquisition and performance requirements in young, awake children (Supplementary file: Interview guide). All interviews were done by MH, whose clinical experience with UGVA in pediatrics allowed her to probe nuances in participants’ responses.

Data collection and analysis proceeded iteratively. Initial sampling included four participants from various pediatric departments in a single center (pediatric anesthesiologists, emergency, and intensive care physicians). Open coding (MH, JM, JF) of the first four interviews revealed consistent stepwise approaches. Responses regarding complexity factors and practice variations were more diverse and harder to articulate. Hence, subsequent data collection and analysis aimed to elaborate our understanding of these features, including a theoretical, purposeful sampling of experts from different backgrounds and settings, including nurse practitioners from specialized vascular access teams with experience in pediatrics and neonatology, both in and outside the Netherlands [29].

During data analysis, the team grappled with multiple orientations to complexity [11]. Compatible with our incident-based technique, our analysis began with a cognitive science orientation towards complexity as a characteristic of a task, where the level of complexity is determined by the amount and integration of knowledge, skills, and attitudes required for performing that task successfully [45,50]. Informed by the data, this analytical orientation shifted to include a more sociological, constructivist orientation, defining complexity as “a dynamic and constantly emerging set of processes and objects that not only interact with each other but come to be defined by those interactions” [28]. This shift towards a social science perspective on complexity in medical education also prompted us to focus on adaptability and consider adaptive expertise theory as a more suitable lens for understanding how complexity in technological-enhanced performance may be navigated [2,11,51]. We ceased data collection after interviewing 11 participants from variable pediatric contexts when no new insights were evident regarding the patterns we were identifying, suggesting data sufficiency [29,52]. (Supplementary file: Table)

## Results

The first section of the results describes four intertwined domains of complexity when performing UGVA in young and awake children: conceptual, psychomotor, contextual, and educational. The second section reports the key processes that experts use to navigate among these intertwined complexities to achieve successful performance.

### A) Complexity domains

#### Conceptual complexity: conceptual refinement for safe implementation

Participants described how concepts of UGVA need to be refined when performing in young and awake children. First, they recognized a critical distinction between “ultrasound-*guided* vascular access” (UGVA) and “ultrasound-*located* vascular access” (P3). Ultrasound guidance of the needle tip towards small blood vessels in deep subcutaneous tissue requires dynamic and precise visualization and control over the needle tip, assuring correct needle placement without vascular damage. Our participants stated that while practitioners might “claim to perform UGVA” (P1), in reality, they often use ultrasound to “locate” the blood vessel and needle but then proceed by “poking until the vessel is hit” (P1), potentially causing vascular damage in subcutaneous tissue. While ultrasound-*located* vascular access may be a valuable improvement over traditional “blind” techniques, disregarding conceptual precision was reported to lead to a “false sense of security” of correct needle placement without vascular damage and “delayed detection of complications” (P1).Participants underscored the challenge and stakes of integrating UGVA safely in pediatrics. As one participant described “where multiple attempts to IV access used to be acceptable, the demand is increasingly developing towards first pass success” (P4). They argued for vascular access policies in pediatrics to ensure appropriate and safe implementation.

#### Psychomotor complexity: dealing with sensory challenges

Performing UGVA required integrating visual, tactile, proprioceptive, and cognitive processes. In describing how sensory inputs translate into precise hand movements, participants emphasized how misalignments of sensory inputs create confusion. As one participant stated, “You think you see what you’re seeing or feel what you’re feeling, but it doesn’t always add up” (P1). Secondly, sensory distortions were described: “Every movement I make with the needle will be enlarged several times on the ultrasound screen. It can look and feel like you’ve bridged a large distance, but in reality, it will only be a millimeter or so.” (P9). Thirdly, while tactile feedback plays a role, “tactile input is not something you can count on” (P1). Many struggled to describe their learning trajectory, stating that “something clicks when it [sensory inputs] all fits” (P6). Prior experiences, such as working with microscopic samples (P9) or gaming (P10), were considered to promote the development of the required psychomotor abilities. A constant search for sensory control was described as crucial for successful performance: “I will only move forward if I know what I see and what I don’t see.” (P9). However, when “control is lost” (P4), for instance when a child moves, participants described that “a few crucial moments” (P8) in between the child’s movements were “somehow enough” (P8) to quickly align sensory inputs and allow advancement of the needle. Mastery of this kind of rapid adaptation characterizes UGVA in children as “tricky to learn but simple once you master the crux of the procedure: finding that needle tip and keeping it in sight” (P6). Descriptions of mastery, however, were consistently followed by a notable caveat: “as long as the arm lies still” (P6).

#### Contextual complexity: dependence on child compliance, ergonomy, and team support

Beyond technical proficiency, participants stressed the importance of managing contextual factors, particularly ensuring child cooperation. “Even one muscle spasm” (P9) disrupts the precise coordination required for UGVA. Consequently, when a child resists the procedure, “forcing the arm to lie still” (P8) will not work. Applying physical restraints “against someone’s will” (P7) was also viewed as not in line with “the kind of care you want to provide” (P8), necessitating strategies to gain voluntary compliance. Super users described an “emotional charge” (P6) when they “get called in, yet again, to a child in distress after several failed attempts” (P2). As one participant described the high stakes involved—“where multiple attempts to IV access used to be acceptable, the demand is increasingly developing towards first pass success” (P4)—others emphasize how when they get called in that “everyone wants to get it over with quickly, but [UGVA] just doesn’t work that way.”P5). They needed “time to set the scene” (P5), because “you first want the child calm and comfortable before you can even think about performing UGVA” (P1). This “setup” required “ergonomic” organization (P5) of the ultrasound, the materials, the child, and the supporting nursing team, to facilitate the expert’s hands in executing precise and subtle movements. Participants also highlighted how a supporting team member acted “like my own third hand” (P2), responding in sync to maintain control when a child moves unexpectedly. Hence, procedural success depended on “fostering comfort and trust” (P4) and “creating the optimal setting” (P8), despite high-pressure circumstances: “a whole lot of fuss that might require more time than the procedure itself, but crucial for success” (P6).

#### Educational complexity: grappling with tacit and moral dimensions

Mastering this procedure was described as “constant refinement” (P5) that is “still ongoing, even on expert level” (P9). Education was therefore emphasized as a fourth complexity. Two major challenges were delineated: 1) the tacit and 2) the moral dimension of learning UGVA in children. First, the precise psychomotor skill was described as tacit, necessitating an internal connection between cognition and hands. “Simulation-based training does not suffice for performance in pediatric practice” (P1) and trainees reported feeling mostly “on their own” (P1) due to the challenge that both teacher and trainee faced verbalizing and understanding guidance of these tacit processes. Hence, mastery was described as effortful, where an “invisible threshold” (P5) needed to be conquered: “It felt like an extra neuron was connected that made the link between knowing what I had to do and being able to really execute it” (P8).

As a result of this tacit nature, participants viewed bedside practice on children as necessary or inevitable. However, they characterized learning to perform UGVA on awake children as a “difficult [moral] choice” (P6) that required them to “shut out their own emotions” (P7). One participant shared their dilemma that “children are not good ‘test subjects’, but this is what you need to learn to deal with” (P4), underscoring the morally charged context of learning pediatric UGVA.

### B) Navigating complexity

In the sections above, we have separated these complexity domains for clarity of presentation, but in real-life performance, participants depicted them as intersecting and interdependent. Their stories of navigating these complexities for a successful performance drew on recurring metaphors clustered around theatre performance. We did not prompt for these metaphors and were struck by their prominence in the data.

First, UGVA in young and awake children was described as a “choreography of efficient, subtle and smooth hand movements” (P5) amidst a careful “orchestration” (P6) of co-actors to “act and react synchronously” (P8) to unexpected changes. Repeated descriptions of meticulous and strategic coordination of co-actors and materials were given that supported successful performance, such as positioning the ultrasound machine “in the line of sight” (P5), or creating a “soothing atmosphere” (P4). In addition, this way of framing performance benefited the participant’s role as a teacher by “fine-tuning their choreography and orchestration” (p5). In performance, collaboration was considered the key, not only for the coordinating aspects but also for achieving the required trust, compliance, and engagement of the child and parent. Participants highlighted that the true expertise for procedural trust and comfort lies with the other actors in the performance: team members (e.g., child life specialists and nursing staff) and the parents.

Second, participants described a synergy or “some kind of magic” (P8) that happens in a performance when all dynamic complexity factors “align” (P11), “fit” (P6), or “coincide together” (P8). Participants struggled to define this phenomenon yet emphasized that child and parent engagement stands at its center. An interdependent relationship between predictability, confidence, and trust was suggested to allow for “[magical] control over the [inherently] uncontrollable” (P8) in pediatric UGVA. As one participant described, “[the ultrasound and mastery of psychomotor skills] give me confidence and courage to state to a child and parent: ‘I will only need one attempt’. I can’t guarantee that with traditional [blind] vascular access, but I can with ultrasound.” (P5). Such predictability fosters a child’s trust and compliance to voluntarily lie still (P10), which in turn allows for “psychomotor control” (P8) required for successful needle placement. This reinforcing cycle was attributed to the ultrasound: “the ultrasound gave me confidence and gave the child trust. And there was some kind of serenity that enabled me to do as I predicted” (P10).

Lastly, participants reflected on “star qualities” (P4) that contributed to successful performance and emphasized these as “basic qualities that do not necessarily have to do with UGVA” (P4) but may facilitate skills development, such as “critical self-reflection” (P6), “problem-solving” (P1), “creativity (P4), persistence (P4), gentleness (P9) Among the key attributes mentioned, “precision” and “courage” stood out across the data. Precision was described as both an attitude—“being precise in every step” (P4)—and an aptitude, linked to psychomotor skill development or “muscle tone regulation” (P4). Courage encompassed addressing moral challenges, such as managing the “inherent unpredictability of young children” (P7) whilst “not being afraid to cause harm” (P7, P9). Courage also involved “deciding when to say ‘stop, we are not doing this’” (P6) when complexities became insurmountable.

## Discussion

Grounded in established expertise theories, this qualitative study examined the complexity of expert performance involving a technology-enhanced procedure in a high-stakes clinical context [33]. Zooming in on UGVA in young, and often non-compliant children, we uncovered experts’ largely tacit, conceptual understandings that underpin successful performance in a demanding context. We offer rich descriptions of four distinct complexity domains: conceptual, psychomotor, contextual, and educational. Importantly, these complexities intersect dynamically in real-world practice, and the novel contribution of our work lies in articulating how experts strategically navigate these intersecting complexities to maximize precise motor skill performance. We found that our experts’ deep conceptual knowledge resonated strongly with the principles of adaptive expertise — a theoretical framework within expertise theory that emerged inductively during our analysis, rather than shaping our initial research design. As such, the empirical data of this CGT study does not aim to generate new theory, but instead contributes constructively by elucidating relational dynamics and key influences that shape expert performance as a complex social phenomenon in real-world pediatric practice [29]. In the following sections, we explore adaptive expertise theory in relation to emerging technologies, motor learning, and transfer theory. Next, we present three insights with potential implications on instructional design.

### Adaptive expertise theory

By recognizing our ‘super-user’ participants as adaptive experts, we underscore the assertion within technical medicine that cognitive adaptability and efficient expertise development are essential for health care professionals to thrive in a future with emerging medical technologies, let alone to improve patient care [2,51]. While routine expertise in a specific domain offers significant advantages, including rapid knowledge retrieval, effective self-monitoring, and efficient performance, it is increasingly viewed as insufficient preparation for healthcare’s exponential changes in complexity [2,33,51]. The model of adaptive expertise conceptualizes an optimal corridor between routine and adaptive expertise [53]. Routine expertise requires deliberate practice for efficient mastery of procedural (*how to*) knowledge, whereas adaptive expertise demands the same level of mastery while integrating deeper conceptual (*why*) knowledge that enables innovation in uncertain or complex situations [53,54]. While the theory-informed contrast between ‘routine’ and ‘adaptive’ expertise offers conceptual clarity, it risks being misinterpreted as a linear or hierarchical progression from one to the other. Increasingly, scholars argue that these forms of expertise must always be viewed as intertwined [22,53]. Yet, how this interdependence manifests in real-world clinical practice remains insufficiently understood [22,53].

From a motor control and learning perspective, Bernstein explained early on how repetitive practice of routine skills actually does not involve repetition at all [20]. Contemporary research continues to show that adaptability is central to coordinate and control movement within an inherently noisy human motor architecture [20]. Bernstein’s work remains influential and relevant, suggesting that routine and adaptive forms of motor skill expertise reflect progressions along the continuum of developing dexterity [20,22]. Although our study was not explicitly grounded in motor learning theory, the findings – emerging from real-world expert performance – invite reflection through this evolving body of work. We focused our study to grasp complexity of real-world clinical performance as a whole, and our findings emphasized how motor control expertise is intertwined and influenced by cognitive, social, and contextual aspects. As such, our findings may enrich both contemporary motor learning and expertise theories by empirically illustrating how social, environmental and moral considerations are not peripheral but integral to motor performance in high-stakes clinical contexts.

The strong conceptual knowledge that our participants expressed across different domains of complexity prompted us to revisit our initial account of transfer [17]. Cheung et al outline various models of transfer and their relation with adaptive expertise [17]. And while the cognitivist account focuses more on memory and information processes, our findings align more closely to the described constructivist account, which sees learning and transfer as making meaningful associations that prepare learners for future learning and problem-solving [17]. Concepts in the data, like ultrasound *located* vs *guided* vascular access, sensory distortions, ergonomy, and use of theatre metaphors, suggest our experts’ capacity to construct conceptual and meta-cognitive strategies [55]. Based on this account, we further reflect on some of these meta-cognitive strategies within this situated perspective and discuss how they could potentially inform future instructional design [17].

### Potential implications for instructional design

We present three insights with potential implications on design for UGVA in pediatrics specifically and procedural skill training generally: 1) rethinking routines in psychomotor skills and the role of simulation to foster adaptive expertise, 2) navigating moral tensions and training for high-stake adaptive performance, and 3) addressing knowledge integration and socio-material influences in adaptive expertise by unraveling tacit complexities that shape real-world adaptive expert performance.

A first instructional implication concerns the exceptional psychomotor precision and challenges involved in UGVA in young children and neonates. Although expert performance may appear smooth and routine, our participants emphasized that successful performance demands constant, fine-tuned adjustments to ambiguous and often confusing sensory-motor and tactile cues, especially when unexpected movements occur during the procedure in an awake child. Participants’ emphasis on learning how to navigate sensory challenges reinforces the view that motor control expertise involves developing effective strategies to interpret and adapt to the sensorimotor perceptions during movements [56]. However, current UGVA training usually relies on static phantoms that prioritize routine execution over perceptual-motor adaptability – an approach our participants described as insufficient preparation for real-world performance.

Drawing on motor control theories, scholars advocate for motor learning simulations that encourage the exploration of motor redundancies and strengthen movement precision and stability [22,56,57]. While deliberate practice – effortful, goal-directed, and feedback-driven – is an established design feature in expertise development [32,58], motor learning scholars also argue that it should be complemented by deliberate play – encouraging low-stake experimentation of movement patterns and creative strategies to unexpected challenges [56,59,60,61]. Our findings echoed the value of these different forms of practice as contributing to their motor control expertise: participants described both effortful refinement of skills – while noting that giving and receiving feedback on precise motor actions is very challenging – and earlier experiences of exploratory practice such as gaming or microscopic interventions [12,32]. As a result, we suggest to further invest in dynamic simulation designs that foster adaptive expertise [57] and consider including serious gaming for technical skills development in medicine [62]. Nonetheless, our findings underscore an intricate interplay between motor skill expertise and perceptual, cognitive and contextual influences. Therefore, while deliberate part-task practice can improve foundational psychomotor skills, it remains insufficient to prepare learners for the full complexity of UGVA in real pediatric contexts [63].

A second implication relates to the critical role and associated stressors of work-based training in cultivating adaptive expertise for high-stakes clinical contexts [51]. Participants extensively described mounting expectations for first-attempt success, the challenges of managing the unpredictability of a young child’s cooperation and their reliance on socio-material factors—illustrating the whole task they faced in practice. While productive struggle is considered a key feature for developing adaptive expertise, our study revealed moral and practical tensions that arise in the pursuit of pediatric procedural excellence [64,65,66]. Notably, participants voiced ethical concerns about necessary practice on vulnerable patients in inherently high-stakes pediatric practices. Educational design often overlooks this moral dimension despite the growing attention to ethical considerations for all involved in pediatric procedural practices [38,67].

The question of how the pursuit of excellence conflicts with the pursuit of a learner’s well-being in medical education presents an ongoing scholarly debate [68]. Research from other high-pressure domains such as competitive sports, military, and law enforcement, affirms how achieving excellence inevitably involves variable degrees of discomfort, stress or anxiety. To mitigate the impact of stress or unexpected events on perceptual-motor performance, research in these fields highlights the important constraint-led training and role of coaching and teamwork [61,69,70,71]. Although our findings may stem from a different high-stakes context, our participants similarly emphasized the importance of team coordination in alleviating constraints that compromise psychomotor performance, along with the need for a guiding teacher throughout their learning trajectory.

Emerging medical education research points to promising authentic team-based strategies to prepare for high-stakes performance – such as stress exposure simulation [71], constraint-led perceptual motor learning [72] and work-based learning elements that foster adaptive expertise [51]. Procedural skills training for UGVA in children could potentially benefit from these strategies. Therefore, rather than progressing linearly from simulation to practice on real patients, we advocate for representative whole-task training that prepares for high-stakes performance. We argue for a staged approach that intertwines part- and whole-task training within simulated scenarios of increasing complexity. Instructional design models – such as the Four-Component Instructional Design (4C/ID) model [26,63,73] — can allow such design by aligning learning tasks with the complexity of real-world performance.

A third and last implication centers on the way knowledge and strategies could be represented in training. Through theory-informed inquiry, this study sought to elicit tacit understandings of how experts navigate real-world complexity. Our participants expressed deep conceptual knowledge of how to adapt to uncertainty and high-stakes situations while optimizing precise motor skill performance. In doing so, our empirical work may help to address two notable gaps within the field of adaptive expertise: 1) we offer insights into tacit knowledge integration illustrating how our adaptive experts represented complexity in high-stakes performance [74,75], and 2), we highlight the, often overlooked,- role of socio-materiality and collaboration as key conditions that shape adaptive expert performance [33,48,75].

Knowledge integration and representation in the development of adaptive expertise warrants further attention to better understand how experts deal with novel situations [74]. In our study, theatre metaphors emerged as a way for participants to articulate their deep conceptual grasp of the nuanced and adaptive demands of successful performance: *1) UGVA was compared to performing a smooth and precise “choreography”, supported by certain individual “star qualities”; 2) to execute the choreography successfully*, *an “orchestration” of the socio-materiality of the performance was required through collaboration and coordination; 3) when all dynamic factors “align”, a synergic effect can occur when the engagement of child and parent can result in “some kind of magic” performance*. While metaphors should be used with caution as an educational resource [76,77], they may provide valuable insight into how clinical experts represent and integrate their tacit knowledge in complex clinical performances. To allow for transfer and adaptability in complex and uncertain contexts, information processing theory in motor learning highlights that individuals need to construct meaningful associations or meta-cognitive schemas for each step of a procedure and interdependent interactions [17,55,78]. Comparable metaphorical language has been reported previously in relation to high-performing resuscitation teams or surgical teams [79,80]. As such, we consider this lens of performance arts to potentially help learners grasp complexity holistically—to conceptually simplify without being simplistic [81,82]. Given that psychomotor learning depends on trainees’ internal cognitive processing and motor control, one participant noted the difficulty that teachers face in providing effective psychomotor instruction. Observing the trainee’s choreography and orchestration of performance was considered a useful way of providing feedback. This suggests future research could explore how metaphors help trainees and teachers develop a shared mental model to support effective feedback in practice [57,83,84].

The mental model of theatre performance also allowed our participants to express the dynamics and subtle affordances of the socio-material environment [56,79]. While our findings acknowledge individual “star qualities”—such as critical self-reflection, precision, creativity, and courage consistent with adaptive expertise literature—our data guided us to move beyond the individual [53,74]. Participants described how ‘orchestrating the right conditions’ or ‘atmosphere’ for a child’s cooperative engagement could only be achieved through teamwork and collaboration with parents. This underscores the vital role of socio-material elements in shaping successful performance—a long-standing connection posited by theorists, yet often underexplored in motor control, educational design, and expertise theory [48,85,86].

In motor control theory, the ecological dynamics framework—and its constraints-led approach for representative training design—emphasizes that skilled perceptual-motor performance can only emerge through dynamic interactions between the individual and their environment [72,87,88]. Developed as a response to individual- and cognition-focused motor learning theories, this framework and associated training design has been applied in fields like sports, information technology, music, and increasingly in medicine, showing promise for enhancing clinical teaching and skills transfer [10,72,89,90,91].

What stands central in ecological dynamics is the creation of representative learning environments that invite learners to perceive and act on the environment’s affordances and constraints. Our study explored, illustrated, and partly elucidated such connections: for example, participants emphasized the meticulous positioning of a team member to enable precise motor skill performance—not merely for ergonomic efficiency, but to function “like their own third hand” and respond synchronously to unexpected movements [42]. This concept of motor synergy or team synergies explains how perceptual-motor performance can emerge across individuals – as seen in domains such as puppetry, or team sports like synchronized swimming —where “reading bodies within a dexterous team” enables collective adaptability [79,92]. Therefore, explicitly eliciting *why* the meticulous positioning of a team member was emphasized as crucial for success, allowed us to understand *how* a physical and mental interaction between team members and environment need to occur.

This kind of explicit connection between procedural and conceptual knowledge is lacking in current training but may enhance the novices’ cognitive integration and support effective team collaboration and synergy. As such, our work responds to a call for adaptive expertise research to attend to the socio-material dimension of real-world expertise [48] and the collaborative dynamics that underpin it [75]. Building on this study’s insights into how and why adaptability is shaped by multiple interactions (technology, perceptual motor, socio-material including both team members and the patient with their caregiver), future research could unravel how the ‘magic’ of successful technology-enhanced performance in complex contexts unfolds [82,93].

### Limitations

Our research design has both affordances and limitations. By using the CGT methodology, we were able to draw on multiple concepts from both cognitive and social science relating to expertise and complexity theories. This allowed us to explore epistemological tensions and illustrate a plurality of perspectives during our iterative approach. Such a theoretically grounded, pluralistic, and flexible study design is considered both a strength and a necessity in complexity research and medical education [11,28]. Nevertheless, a limitation of exploring the intersections of two fields is a necessary partial commitment to either one. For instance, the incident-based interview that sensitized this study to improve interview dialogue stems from cognitive task analysis [45]. Yet, we chose not to conduct a cognitive task analysis, because our early data analysis guided us to seek a deeper sociological understanding of the interconnected complexities of contextual performance [28,29]. While our study was grounded in established theories of expertise, it did not explicitly incorporate motor control or learning theories in the study design. These perspectives increasingly informed our understanding of expert performance. In retrospect, motor learning theory provided a valuable lens to deepen our insights into how complex learning and performance unfold—highlighting that such processes cannot be neatly divided into cognitive, psychomotor, or contextual domains. We argue that an integrative framework such as expertise theory remains well-suited to accommodate this complexity. While our purposeful sample provided rich and sufficient information power to represent the phenomenon under study [94], we recognize the limitations of exploring individual expertise by interviewing only one involved member of the UGVA collective performance. The emphasis on the collective in our findings sharpened our attention to a plurality in accounts of transfer influencing training, as described by Cheung and Kulasegram [17]. This prompted us to reconsider transfer not only as an individual cognitive process, but also as a construction of conceptual associations that help grasp, navigate, and adapt to complexity. Moreover, our data suggest that performance and transfer are afforded by multiple interactions and relationships – dimensions that our individual-focused approach could not fully capture.

These reflections lead us to argue that future research should not only explore *team adaptive expertise* [95] but also extend the notion of *collective adaptive expertise*, to include the dynamic contributions of patients and their caregivers as co-actors within the team [85,96]. ‘Close to practice’ qualitative methods, such as video-reflexive ethnography, can support this aim [97]. Video-based research methods not only illuminate and explore collaboration and the socio-material interactions in authentic healthcare settings, but may also empower healthcare practitioners to refine education and practice from within [48,98]. We advocate for moving expertise inquiry,-like ours,-beyond a cognitive focus on the individual [33], and beyond the motor skill focus of the procedure [42], towards a broader focus on collective synergic performance. This shift may be crucial in demystifying the perceived ‘magic’ in expert performance [99] and in preparing future adaptive experts to navigate the inherent complexities of clinical practice [55].

## Conclusion

In this age of rapid technological innovations in complex health care, effective training is more critical than ever. This study contributes a situated perspective of adaptive expertise in high-stakes, technology-enhanced pediatric practice, by unpacking the intricate interplay of conceptual, psychomotor, contextual and educational constraints involved in ultrasound-guided vascular access in young—and often non-cooperative—children. By eliciting expert’s tacit conceptual understanding, precise psychomotor skill and the socio-material orchestration required for successful performance, we illustrate how adaptability emerges not solely from the individual expert, but through dynamic coordination and collaboration with the environment, team, child and parents. Our understanding of this collective adaptive performance may contribute empirically and theoretically to the field of adaptive expertise research. Participants’ metaphors of theatre performance offered a compelling lens to grasp complexity and adaptability, revealing how experts choreograph precise actions within unfolding clinical realities. By drawing parallels outside of medicine, this study elaborates on perceptual-motor learning with the aim of advancing transfer of new technical skills into high-stakes contexts.

Our work reflects on transfer theories and presents implications for instructional design for pediatric UGVA specifically and procedural skills in medicine generally: from decontextualized skill acquisition to dynamic, whole-task learning environments that foster 1) perceptual-motor flexibility in deliberate practice and play, 2) moral readiness for high-stakes performance and 3) conceptual knowledge integration with affordances and constraints of the environment to support clinical workplace training. Future research should further explore the relational dynamics and socio-material interactions that underpin the synergy that occurs in collective adaptive performance—preparing future adaptive experts not only to master technical skills, but to understand what harnesses ‘the magic’ in collective expert performance.

## Additional Files

The additional files for this article can be found as follows:

10.5334/pme.1798.s1 Supplementary File. Table theoretical sampling and iterations.

10.5334/pme.1798.s2 Supplementary File. Interview guide.
