# Supplementary material for: “Some Kind of Magic?” How Adaptive Experts Navigate Complexity in Pediatric Ultrasound-Guided Vascular Access
Source: Perspect Med Educ. 2025 Nov 12;14(1):773–86. doi: 10.5334/pme.1798 (PMC12617406; doi:10.5334/pme.1798)
Supplement: Supplementary File. — Table theoretical sampling and iterations. [file pme-14-1-1798-s1.pdf]

Supplementary table 2: Overview on how data informed theoretical sampling, alterations in interview guide, and proposed definitions of complexity.

	**Sampling**	**Interview guide**	**Saturated data**	**Changes/further focus**
Interview 1-4	P1: Ped anesth<br>P2: Ped anesth<br>P3: PICU/PEM<br>P4: ICU<br>Within one center in the Netherlands	1. Priming memorable incident<br>2. Timeline / stepwise approach/task inventory<br>3. Deepening – Variation and complexity<br>4. Skills acquisition what if queries	Task inventory	- Less focus on task inventory<br>- More focus on variation in practice and skills acquisition
Interview 5-7	P5: Ped anesth / PICU<br>P6 and P7: Nurse specialist in vascular access teams	1. Priming<br>2. Complexity<br>3. Variation in practice<br>4. Expertise development	Variation in practice<br>Current skills acquisition	- Introduction concepts such as choreography, ergonomy, theater<br><br>- Became clear that awake and young children pose the greatest complexity
Interview 8-11	P8: PICU and PROSA MUMC+<br>P9: NICU<br>P10: Ped Vasc access team nurse specialist<br>P11: NICU	1. Priming<br>2. Complexity – what do you need for success?<br>3. What if 'child moves'<br>4. Expertise development	Conceptual patterns (choreography, coordination, collaboration, conceptual precision, …)	- More focus on theatre analogies<br>-Identification of remaining tacit dimensions

P(n#): Participant (number #)

PICU: Pediatric Intensive Care Unit

NICU: Neonatal Intensive Care Unit

Ped anesth: Pediatric Anesthesiology

PEM: pediatric emergency medicine

PROSA: procedural sedation and analgesia unit
