# Supplementary material for: “Some Kind of Magic?” How Adaptive Experts Navigate Complexity in Pediatric Ultrasound-Guided Vascular Access
Source: Perspect Med Educ. 2025 Nov 12;14(1):773–86. doi: 10.5334/pme.1798 (PMC12617406; doi:10.5334/pme.1798)
Supplement: Supplementary File. — Interview guide. [file pme-14-1-1798-s2.pdf]

**Supplementary information: Interview guide**

**Intro**

1. Thanks for your time
2. A bit about myself and this project:
    a. I'm a PhD candidate researching UGVA skills
    b. **Goal of research**: My colleagues and I investigating how we can create better trainings for UGVA, so more people will start using it. We're reaching out to experts to understand how professionals do UGVA in practice.
    c. **Reassure** expert that we are not evaluating their work, but just curious about how they work
    d. **Check** informed consent, ask if OK to record. Repeat that data will be stored / processed anonymously.
    e. Any questions before we get started?

**Eligibility check / characteristics of experts background etc.**

3. Please introduce yourself
4. How long have you been using UGVA in children and neonates?

PART 1 based on Incident based interview techniques (Cognitive task analysis (CTA) methods)

**Priming Memorable incidents** (cfr Critical decision method)

5. What's **the most memorable example** you've experienced? **What made it memorable?**

**Skills Hierarchy – stepwise approach** (cfr Timeline – CTA)

6. Can you describe the example again. Can you **break down the task in 3-6 steps**?
7. For each step, deepen what skills / knowledge are needed to perform this step.
    a. For each skill, ask what other skills are necessary to perform the overarching skill
    b. For each skill, ask which constituent skills are necessary (i.e., go deeper)
8. Identify the 3-5 skills that should be given the most priority in a UGVA training.

**Task Inventory – variability** (deepening by asking what if questions cfr CTA)

9. I want to learn how UGVA **varies** between different patients or situations. Could you give a few examples of UGVA applications that felt very different? Based on answers, prompt for differences between:
    c. Patients
    d. Tools
    e. Time schedules
    f. Staff/support
    g. Parents/caretakers
10. Which variants are most common? If you were to teach a class on UGVA, what's **the most important task to focus on**?

**Complexity** Matrix (deepening by probing – look for action verbs that tells you critical decisions are made)

11. What's the easiest UGVA you've ever done? **What made it easy?**
12. What's the hardest UGVA you've ever done? **What made it hard?**
13. So far, I'm hearing that ___ makes it easier and ___ makes it harder. Are there any other factors that make UGVA easier or harder? [Based on answers, figure out ordering of complexity]

PART 2 of the interview: skill and expertise development

Skill **acquisition**

14. How did you acquire the UGVA skill? How were you trained?
    h. Did the training program involve working on real-life tasks?
    i. Did the training program include support and/or feedback?
    j. Did the training program include demonstrations?
    k. Did the training program include sufficient opportunities for practice/application of knowledge?
    l. Were you able to integrate the learned skill in your daily practice?

**Expertise** development

15. How did you develop your expertise after training, in the workplace?
    m. Did you have a trainer or expert around? Who?
    n. Did you set specific goals for your training? How did you judge quality of performance?
    o. Did you request feedback on your performance? From whom?
    p. Were you able to practice repeatedly? Which activities helped you?

(added question after interview 5)
**Can you give us insights in why you succeed in performing UGVA in awake children, despite the complexities that you reflected on? What is needed to become successful?**

**<u>Close</u>**

Thank you

Would you like to be involved in further research relating to this subject

Can you refer to other performers in your network that you consider very successful or expert in UGVA in young children or neonates?
